# Pediatric disability weights following injury based on patient-reported data from a multinational cohort

**DOI:** 10.1007/s00431-026-06845-2

**Published:** 2026-03-21

**Authors:** Joanna F. Dipnall, Frederick P. Rivara, Shanthi Ameratunga, Fiona E. Lecky, Ronan A. Lyons, James E. Harrison, Belinda J. Gabbe

**Affiliations:** 1 School of Public Health and Preventive Medicine, Monash University, Level 3, 553 St Kilda Road, Melbourne, VIC 3004 Australia; 2 Institute for Mental and Physical Health and Clinical Translation, School of Medicine, Deakin University, Geelong, Australia; 3 Departments of Pediatrics and Epidemiology, and the Harborview Injury Prevention and Research Center, University of Washington, Seattle, WA USA; 4 School of Population Health, University of Auckland, Auckland, New Zealand; 5 Centre for Urgent and Emergency Care Research, School of Health and Related Research, University of Sheffield, Sheffield, UK; 6 Emergency Department, Salford Royal Hospital, Salford, UK; 7 Population Data Science, Swansea University, Swansea, UK; 8 Flinders Health and Medical Research Institute, Flinders University, Adelaide, SA Australia

**Keywords:** Disability weights, Health-related quality of life, Trauma, Injury, Children, Pediatric, Adolescents, DALY, YLD

## Abstract

Empirical data on post-injury disability in children is limited and deriving disability weights is urgently needed. The aim of this study is to utilise pooled data to determine adequate disability weights in children and adolescents. Five longitudinal prospective cohort studies of pediatric injury survivors aged 5–17 years were pooled (*N* = 1972) to create case-based pediatric weights for established nature-of-injury classifications using four types of EQ-5D utility scores to represent overall health-related quality of life. Healthy population norms from seven countries formed a sensitivity analysis. Disability weights (DW) were calculated at 1 month, 4 months, 6 months, and 12 months post injury to produce two disability weights per injury class: 12-month residual disability weights (12dw) and 12-month annualised weights (12aw). DW for asphyxiation/non-fatal submersion, spinal cord lesion, fracture of the femur, fracture of pelvis, and fracture of vertebral column, were more than twice that of the lowest DW for fracture of clavicle, scapula, or humerus, and fracture of radius or ulna. 12dw for moderate/severe traumatic brain injury (TBI) was 19% higher than minor TBI and 25% higher for 12aw. *Conclusion*: Different DW should be applied to DALY calculations for children and adolescents compared to adults. The calculation of these DWs is complex and warrants further investigation. This study confirms that injury is often a chronic disorder and burden of disease for children and adolescents and estimates should reflect this situation.

**Table:** 

What is Known: • Traumatic injuries pose substantial threats to children’s health, education and social inclusion • Much-needed disability weights, used in calculating the years lived with disability (YLDs), has been limited by the lack of empirical data on postinjury disability in children	
What is New: • This study confirms injury is often a chronic disorder and burden of disease for children and adolescents • Injury-group disability weights for children and adolescents are provided for YLD calculations, and these weights differ to those used for adults

**Supplementary Information:**

The online version contains supplementary material available at 10.1007/s00431-026-06845-2.

## Introduction

Traumatic injuries pose substantial threats to children’s health, education and social inclusion, alongside wider impacts on society [1–4]. The limited availability of empirical data on postinjury disability in children has slowed the progress in deriving disability weights used to calculate years lived with disability (YLDs) [5–8], a component of Disability-adjusted life years (DALYs).

Calculating disability weights for YLD estimates from empirical data requires the use of multi-attribute utility instruments (MAUIs). The EQ-5D-3L (EQ-5D), a generic measure of health status, includes five dimensions (mobility, self-care, usual activities, pain or discomfort and anxiety or depression) and is a widely used MAUI for the calculation of disability weights [9, 10]. However, EQ-5D adaptations for children and young people are at varying stages of development (https://euroqol.org/) with different component dimensions and values, challenging interpretations [11].

Cohort studies of injured children and adolescents have generally involved relatively small samples, recruited at the interface with healthcare, and have been limited to short periods of follow-up and focused on particular types of injury (e.g. traumatic brain injury) [12–14], thereby commonly omitting cases of injury from certain categories (e.g. victims of child abuse, self-harm injuries) and children of non-native language backgrounds. This limits the ability to base robust population-based estimates of the burden of childhood injury [15]. Pooled longitudinal data from multiple international sources enables covering a broader range of injuries, improved generalisability, more precise estimates and greater statistical power to identify predictors of Health-Related Quality of Life (HRQoL) following injury.

This study used previously published pooled data from the pediatric Validating Injury Burden Estimates Study (VIBES-Junior) [15] involving injury cohorts from four high-income countries [16] to determine adequate disability weights in children and adolescents. The secondary aim of this study was to compare these disability weights in children and adolescents with the disability weights in adults.

## Methods

### Pooled cohorts

Five longitudinal cohort studies of pediatric injury survivors aged 5 to 17 years old from the VIBES-Junior project [15] (Table 1) were pooled to create case-based pediatric weights for individual injury diagnosis codes and established nature-of-injury classifications. The studies were selected as they: included all injury types; collected outcomes at follow-up to at least 12 months post injury; included standardised measures of function, health status or health-related quality of life instruments; and included an injury diagnoses coding (Table 1). These cohorts were pooled using the Data Integration Protocol in Ten Steps (DIPIT) [17], a systematic ten-step approach that ensured the final pooled data was appropriately harmonised for the analysis (e.g. file formats, links for integration, key variables such as socioeconomic status, mechanism of injury, intent). The Australian cohorts contained pediatric patient data from the state of Victoria. The Victorian State Trauma Registry (VSTR) is a population-based trauma registry that captures data about all major trauma patients [18]. The Victorian Orthopedic Trauma Outcomes Registry (VOTOR) is a clinical registry of orthopedic injuries, treatment, complications and outcomes based on admissions to four Victorian centres [19]. The US Children’s Health After Injury (CHAI) included children with mild, moderate and severe TBI or with upper extremity injuries who presented to a set of US hospitals [20], and the United Kingdom Burden of Injury (UKBOI) was a study of injured individuals with children recruited from emergency department (ED) presentations and hospital admissions in four UK centres [21]. The British Columbia Children’s Hospital Longitudinal Injury Outcomes (BCCH-LIO) study included children who attended the British Columbia Children’s Hospital in Canada for an injury [8]. Across all cohorts, children < 5 years of age, died in the hospital or did not meet the inclusion criteria were excluded from each cohort. The VOTOR, CHAI cohorts related to a specific injury type (i.e. orthopaedic and TBI respectively) with exclusion criteria related to these injuries.

**Table 1:** Summary of injury-specific cohort studies included in research^

Study, Setting, Date (month/year)	Inclusion/Exclusion criteria		Participants base^ & age	Post-injury follow-up time points, mode of interview, Completion rate*	Injury diagnosis coding	EQ-5D Measure
VSTR Australia 03/2009 to 03/2017	Inclusion: Victorian hospital admission, ISS > 12, ICU admission > 24 h and requiring mechanical ventilation; urgent surgery (intracranial, intrathoracic, intra-abdominal, or fixation of pelvic or spinal fractures), burns ≥ 20% total body surface area Exclusion: Children < 5 years, died in hospital, did not meet inclusion criteria		*n* = 693 5 to 17 years	6-, 12- and 24 months Telephone Completion Rate: 89%	ICD-10-AM	EQ-5D utility score
VOTOR Australia 03/2009 to 03/2017	Inclusion: All patients admitted with a new orthopaedic (bone or soft tissue) injury with a length of stay > 24 h Exclusion: died in hospital; pathological fracture related to metastatic disease, aged < 16 years; isolated soft tissue injury managed non-operatively; did not meet inclusion criteria		*n* = 431 16 to 17 years	6-, 12- and 24 months Telephone Completion Rate: 91%	ICD-10-AM	EQ-5D utility score
CHAI USA 03/2007 to 09/2008	Inclusion: Presentation to ED or hospital admission for either a TBI (randomly selected) or an upper extremity injury (age-sex matched to mild TBI patients) to the regional children’s hospital; the one Level 1 trauma centre, one of four Level 3 or 4 trauma centres, and three non-trauma centre hospitals randomly selected from all hospitals in King County, Washington, plus one Level 1 trauma hospital from Pennsylvania Exclusion: Children < 5 years; did not meet inclusion criteria		*n* = 579 5 to 17 years	Baseline, 3-, 12-, and 24 months Online, telephone and postal Completion Rate: 98%	ICD-9 mapped to ICD-10	PedsQL scores mapped to EQ-5D utility score Online, telephone and postal
UKBOI UK 09/2005 to 04/2007	Inclusion: Stratified sample of patients by age and injury type presenting to ED or a hospital admission to four UK area hospitals (Swansea, Surrey, Bristol and Nottingham). Injury was to have occurred up to 2 weeks prior to the date of recruitment or within 4 weeks if the patient is admitted to hospital with a serious injury Exclusion: Children < 5 years; unable to give consent without a suitable proxy; unable to complete questionnaires in the future; no address or leaving the UK permanently; patients with stings and foreign bodies in the ear; did not meet inclusion criteria;		*n* = 121 5 to 17 years	1-, 6- and 12 months Postal Completion Rate: 96%	ICD-10	EQ-5D utility score
BCCH-LIO Canada 02/2011 to 12/2013	Inclusion: Presentation to ED with a primary injury diagnosis at the British Columbia Children’s Hospital (BCCH) ED or were admitted to the hospital Exclusion: Children < 5 years; non-English speaking; did not have an address in British Columbia (BC); did not meet inclusion criteria		*n* = 148 5 to 17 years	Baseline, 1-, 4-, and 12-months Postal Completion Rate: 84%	ICD-10	EQ-5D utility score

*VSTR* Victorian State Trauma Registry, *ISS* Injury Severity Score, *ICU* Intensive Care Unit, *CHAI* Children’s Health After Injury, *ED* Emergency Department, *TBI* traumatic brain injury, *UKBOI* United Kingdom Burden of Injury study, *BCCH-LIO* British Columbia Children’s Hospital Longitudinal Injury Outcomes study. ^Participant base is the patients with at least one HRQoL score. * Completion rate refers to those patients with complete follow-up data for this study (refer Supplementary Table s1 for more details)

The primary published work for this study excluded < 3% due to missing data [16], which was considered acceptable for valid estimations [22]. To align with previous injury-based disability weight research [23], and allow for a viable comparison to the adult population, all children and adolescents aged 5 to 17 years with at least one EQ-5D utility score up to and including 12 months post injury per study were included in this study to calculate the disability weights (*n* = 1972). The baseline characteristics of children and adolescents with and with complete follow-up EQ-5D utility scores included in this study are outlined in Supplementary Table s1. A sensitivity analysis was performed excluding the cohort with the largest proportion of missing follow-up data with marginal differences in results.

### Measures

#### EQ-5D utility score

The EQ-5D utility score was used to represent overall HRQoL. This score anchors include 0 (state equivalent to death) and 1 (full health); negative values represent health states regarded as worse than death. Four of the five cohorts collected this measure. The CHAI study collected the Pediatric Quality of Life Inventory (PedsQL) where responses to the PedsQL were mapped to the EQ-5D using the algorithm developed by Khan et al. [24].

#### Demographic and injury characteristics

Demographic characteristics were collected at baseline and included sex, and age group separated into three groups to align with the World Health Organization classification [25] (5–9 years, 10–14 years, 15–17 years). Diagnosis codes were classified using the ICD 10th Revision (ICD-10). The CHAI data were mapped from the ICD 9th Revision (ICD-9) to ICD-10. All diagnosis codes were mapped to the 2013 Global Burden of Disease (GBD) study injury health states using GBD tables that enabled cross-walking from the ICD-10 codes to the health states [10]. The 17 health states, in order of severity defined by GBD, were in line with previous research (Supplementary Table s2) [16]. A two-group mechanism of injury was used to represent the injuries sustained during transport (motor vehicle occupant, pedestrian, or on a motorcycle or bicycle) and non-transport injuries (falls, struck by/against an object or person, and other mechanisms).

### Statistical analysis

Data were summarised using frequencies and percentages for categorical variables. Mean and standard deviations (SD) and median and interquartile range were used for continuous variables. Raw EQ-5D utility scores were used across all the cohorts to construct the utility weights per injury group. Four types of EQ-5D utility scores were produced to conduct a sensitivity analysis for the resulting disability weights to evaluate the consistency of results:

1. Predicted scores from a previously published mixed effects linear regression model that controlled for sex, age, socioeconomic status, transport mechanism, hospital status (Emergency Department presentation only, Hospital admission), comorbidity, injury severity, time and cohort [16]. Consistent with previous research, this model included all children and adolescents with an eligible EQ-5D utility score at 1-, 4-, 6-, 12- and/or 24 months post-injury. However, only predicted scores up to and including 12 months were used in this study. The 17 injury groups were included in the model as separate binary predictors. Predicted scores > 1 were reset to 1
2. Predicted scores from the previously published model in 1, excluding the injury severity score (ISS) to establish if this variable was leading to an over-control in the model and potential relationship to disability. Predicted scores > 1 were reset to 1
3. Predicted scores from the previously published model in 1, excluding the CHAI to establish if the mapped ICD-9 to ICD-10 injury classes and mapped EQ-5D utility score impacted on the predicted results. Predicted scores > 1 were reset to 1
4. Raw EQ-5D utility scores excluding the CHAI to establish if the mapped ICD-9 to ICD-10 injury classes and mapped EQ-5D utility score impacted on the raw results.

The sensitivity analysis included mechanism of injury to further check consistencies.

Consistent with previous VIBES-Junior research, the 3-month outcomes from CHAI were included in the 4-month estimates [16]. Disability weights were calculated at four time points: 1 month, 4 months, 6 months, and 12 months post injury by subtracting the predicted EQ-5D score from a population norm. Due to the lack of published population norms for each specific pediatric cohort population, an initial sensitivity analysis was performed using scores from 1 and 4 with eight population healthy norm measures: a fixed value of 1, representing *full health*; and seven published country-specific pediatric norms: Australia [26], United Kingdom [27], Hungary [28], Indonesia [29], Japan [30], China [31] and Peru [32]. Each country’s population norms had differing age groups and some excluded sex (Supplementary Table s3). Weights calculated at 12 months post-injury were assumed to represent both residual disability at 12 months and the expected lifelong disability [33, 34]. Sensitivity analyses were performed across injury groups and transport mechanism (transport/non-transport) to check for consistency.

A 12-month annualised weight was calculated by multiplying the EQ-5D differences at each time point by a factor corresponding to the length of the period over which the disability weight applied and summed. Thus, 1-, 4-, 6-, 12-month differences were multiplied by 1/12, 3/12, 2/12, 6/12 respectively and summed together to produce a single annualised disability weight, in accordance with previous research [23]. Results were presented as means with 95% confidence intervals across injury group in tabular and graphical form. Where applicable, a comparison of the published adult injury disability 12-month residual and annualised weights [23] were performed using a two-sample Welch’s *t*-test using to compare the means of the two independent groups and to allow for unequal variances and unequal sample sizes [35]. A Bonferroni adjusted α = 0.003 was used to adjust for multiple comparisons. Analyses were performed using Stata version 18.0 (Stata Corp, College Station, TX, USA) within the Monash Secure eResearch Platform (Monash SeRP), which is supported by the Monash eResearch Centre and Helix at Monash University.

### Ethics

The project was approved by the Monash University Human Research Ethics Committee (project #12,311) with a waiver of consent provided with the approval of the data custodians. The study was conducted in compliance with the NHMRC National Statement on Ethical Conduct in Human Research (2007) and the Note for Guidance on Good Clinical Practice (CPMP/ICH-135/95).

## Results

A detailed overview of the pooled cohorts is published elsewhere [16]. A total of 1972 children and adolescents who had an eligible EQ-5D utility score were included in the prediction model, with the following number of respondents at each time point: 255 at 1 month; 702 at 4 months; 1127 at 6 months; and 1972 at 12 months. The mean age of these respondents across the 5 to 17-year-old age group at time of injury was 13.57 years (SD 3.55), with median age of 15 years (interquartile range 11 − 16.5) (Table 2). Most respondents were male (73.3%), and 37.6% sustained transport related injuries.

**Table 2:** VIBES-Junior pooled cohort baseline characteristics

	VIBES-Junior Dataset					
	VSTR	VOTOR	CHAI	UKBOI	BCCH-LIO	Total
*N*	693 (35.1%)	431 (21.9%)	579 (29.4%)	121 (6.1%)	148 (7.5%)	1972 (100.0%)
Age (years) (mean SD)	14.4 (2.5)	16.6 (0.5)	11.7 (3.9)	11.3 (3.0)	10.0 (3.3)	13.6 (3.5)
Age (years) (median p25 p75)	15 (13–17)	17 (16–17)	12 (8–15)	11 (9–13)	10 (7–13)	15 (11–16.5)
Age Group						
5–9 years	34 (4.9%)	0	181 (31.3%)	34 (28.1%)	62 (41.9%)	311 (15.8%)
10–14 years	252 (36.4%)	0	218 (37.7%)	66 (54.5%)	74 (50.0%)	610 (30.9%)
15–17 years	407 (58.7%)	431 (100.0%)	180 (31.1%)	21 (17.4%)	12 (8.1%)	1051 (53.3%)
Sex						
Male	534 (77.1%)	345 (80.0%)	397 (68.6%)	75 (62.0%)	95 (64.2%)	1446 (73.3%)
Female	159 (22.9%)	86 (20.0%)	182 (31.4%)	46 (38.0%)	53 (35.8%)	526 (26.7%)
Transport Status**^**						
Non-transport	303 (43.7%)	254 (58.9%)	434 (75.5%)	111 (93.3%)	113 (88.3%)	1215 (62.4%)
Transport	390 (56.3%)	177 (41.1%)	141 (24.5%)	8 (6.7%)	15 (11.7%)	731 (37.6%)
GBD 2013 Injury Group						
N33, N34 Spinal cord lesion	31 (4.5%)	5 (1.2%)	*	0	*	38 (1.9%)
N19, N26 Fracture of femur	48 (6.9%)	33 (7.7%)	7 (1.2%)	3 (2.5%)	8 (5.4%)	99 (5.0%)
N20 Fracture of patella/tibia/fibula/ankle	42 (6.1%)	137 (31.8%)	2 (0.3%)	12 (9.9%)	7 (4.7%)	200 (10.1%)
N28 Moderate to severe TBI	218 (31.5%)	**	93 (16.1%)	*	*	319 (16.2%)
N37, N17, N18 Crush injury, fracture foot/hand bones	1 (0.1%)	26 (6.0%)	9 (1.6%)	12 (9.9%)	10 (6.8%)	58 (2.9%)
N43 Internal haemorrhage in abdomen/pelvis	183 (26.4%)	**	*	0	5 (3.4%)	195 (9.9%)
N27 Minor TBI	35 (5.1%)	21 (4.9%)	245 (42.3%)	0	13 (8.8%)	314 (15.9%)
N21 Fracture of pelvis	19 (2.7%)	13 (3.0%)	8 (1.4%)	0	*	41 (2.1%)
N42 Severe chest Injury	25 (3.6%)	13 (3.0%)	*	0	*	41 (2.1%)
N8, N9, N10 Burns (including lower airways)	15 (2.2%)	0	0	*	*	19 (1.0%)
N25 Fracture of vertebral column	29 (4.2%)	40 (9.3%)	*	0	0	72 (3.7%)
N35, N36 Asphyxiation/Non-fatal submersion	6 (0.9%)	0	0	0	0	6 (0.3%)
N40, N44 Contusion/open wound	10 (1.4%)	16 (3.7%)	13 (2.2%)	18 (14.9%)	11 (7.4%)	68 (3.4%)
N14 Other injuries of muscle & tendon/other dislocations	*	22 (5.1%)	*	23 (19.0%)	25 (16.9%)	75 (3.8%)
N15 Fracture of clavicle/scapula/humerus	0	21 (4.9%)	35 (6.0%)	9 (7.4%)	10 (6.8%)	75 (3.8%)
N22 Fracture of radius/ulna	0	59 (13.7%)	79 (13.6%)	21 (17.4%)	20 (13.5%)	179 (9.1%)
Other	30 (4.3%)	16 (3.7%)	75 (13.0%)	19 (15.7%)	33 (22.3%)	173 (8.8%)

*SD* Standard Deviation. *n < 5, **n < 10. ^ Missing data: *n* = 26 (1.3%) for transport status

There was variability in the results for the 12-month residual and annualised disability weights per injury depending on the country norms used (Supplementary Fig. 1). Using the Australian norms resulted in consistently lower disability weights compared to using other country norms, often resulting in negative values. Use of the UK and Peruvian norms resulted in consistently lower disability weights than using the Japanese norms, which were consistently lower than using the Chinese, Indonesian and Hungarian norms. There was also variability in the results for the 12-month residual and annualised disability weights per mechanism depending on the country norms used (Supplementary Fig. 2), with the ordering of countries consistent with the injury groups. However, patterns across injury and transport mechanism were consistent irrespective of the population norm used and for both the 12-month residual and annualised weights. Thus, the fixed value of 1 for the healthy population norm was used for this study as this removed any country bias and ensured meaningful disability weights.

**Fig. 1:**
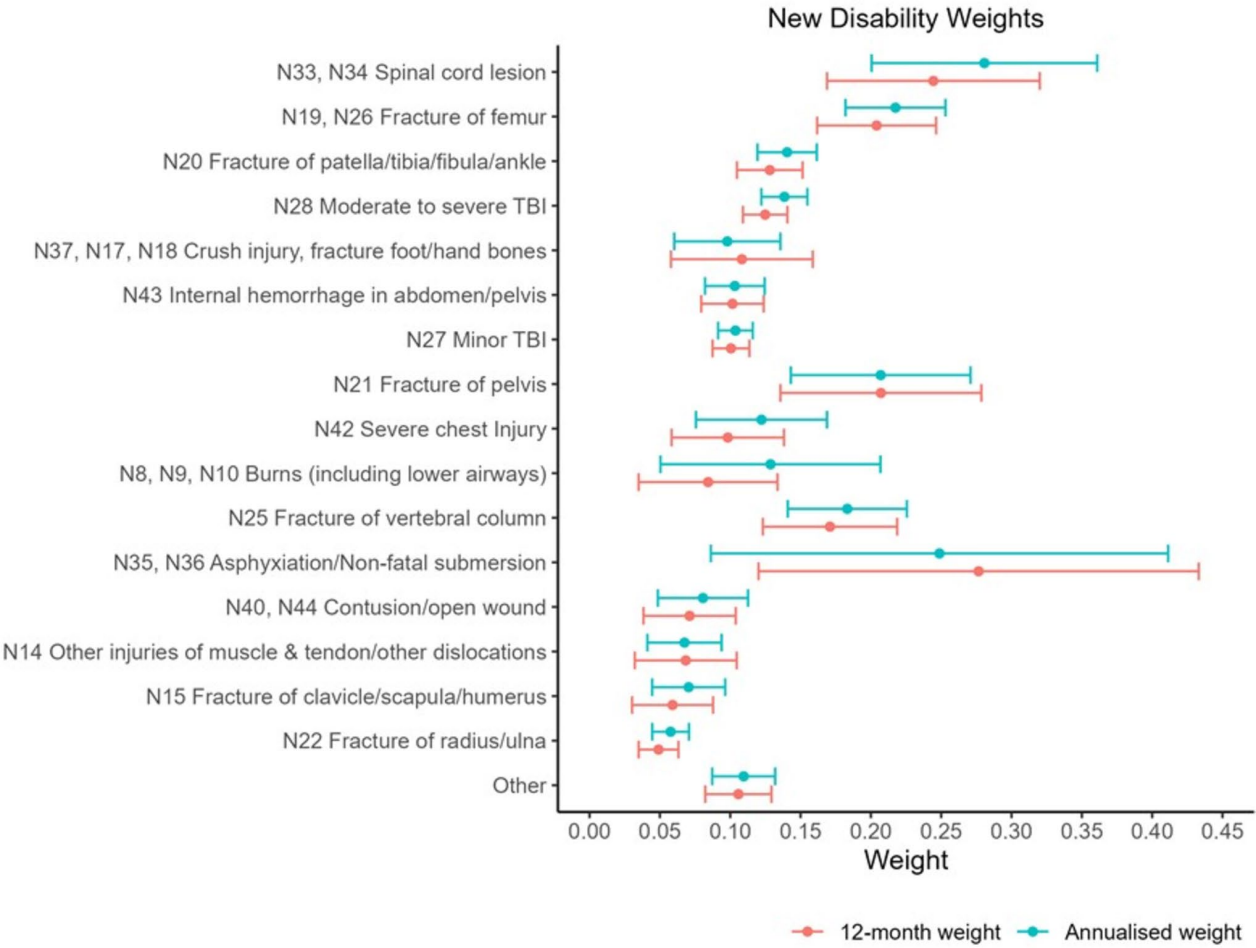
New 12-month and annualised disability weights for children and adolescents 5 to 17 years

The final 12-month residual and annualized new weights for children and adolescents by injury are presented in Fig. 1 and Table 3. The disability weights were highest for asphyxiation/non-fatal submersion, spinal cord lesion, fracture of the femur, fracture of pelvis, and fracture of vertebral column. These injuries’ 12-month residual and annualised disability weights were more than double the lowest disability weights which were assigned to a fracture of clavicle/scapula/humerus, and fracture of radius/ulna. The annualised disability weight associated with asphyxiation/non-fatal submersion was 10% lower than for the 12-month residual disability weight, but the 95% confidence interval was wide and base small for this group (*n* = 6). The 12-month residual disability weight for moderate to severe traumatic brain injury (TBI) (0.125, 95%CI: 0.109, 0.141) was 19% higher than minor TBI (0.101, 95%CI: 0.087, 0.114) and 25% higher for the annualised disability weights (moderate to severe TBI: 0.138, 95%CI: 0.122, 0.155; minor TBI: 0.104, 95%CI: 0.091, 0.116). The disability weights for contusion/open wound and other injuries of muscle & tendon/other dislocations were similar (contusion/open wound: 0.071, 95%CI: 0.038, 0.104; other injuries of muscle & tendon/other dislocations: 0.068. 95%CI: 0.032, 0.105). There annualised disability weight for burns (including lower airways) was 53% higher than the 12-month disability weight, 24% higher for severe chest injury, 19% higher for a fracture of clavicle/scapula/humerus, 17% higher for fracture of the radius/ulna, and 15% higher for Spinal cord lesion.

**Table 3:** New 12-month residual and annualised disability weights for children and adolescents (5–17 years) versus published adult disability weights

Children and Adolescents	Base	12-Month Residual	95% Low	95% High	Annualised		95% High	Adults	Base	12-Month Residual	95% Low	95% High	Annualised		95% High
		Mean			Mean	95% Low				Mean			Mean	95% Low	
GBD 2013 Injury Group								Injury							
N20 Fracture of patella, tibia/fibula/ankle	200	0.128	0.105	0.151	0.14	0.119	0.162	Fracture of patella, tibia, fibula or ankle	3267	0.142	0.132	0.152	0.163	0.154	0.171
*	-	-	-	-	-	-	-	Fracture of hip	2407	0.273	0.259	0.287	0.281	0.268	0.294
N22 Fracture of radius/ulna	179	0.049	0.035	0.063	0.058	0.045	0.071	Fracture of radius or ulna	2316	0.070	0.059	0.081	0.081	0.071	0.091
N28 Moderate to severe TBI^	319	0.125ᵃ	0.109	0.141	0.138ᵃ	0.122	0.155	Moderate traumatic brain injury^	2310	0.186ᵃ	0.172	0.200	0.197ᵃ	0.185	0.210
N25 Fracture of vertebral column	72	0.171	0.123	0.219	0.183	0.141	0.226	Fracture of vertebral column	1550	0.168	0.152	0.183	0.184	0.17	0.198
N42 Severe chest Injury	41	0.098	0.058	0.138	0.122	0.076	0.169	Severe chest injury	1382	0.162	0.146	0.178	0.180	0.165	0.195
N15 Fracture of clavicle/scapula/humerus	75	0.059ᵃ	0.030	0.088	0.070ᵃ	0.044	0.096	Fracture of clavicle, scapula or humerus	1289	0.142ᵃ	0.126	0.159	0.153ᵃ	0.138	0.168
N19, N26 Fracture of femur	99	0.204	0.162	0.246	0.217	0.182	0.253	Fracture of femur	1078	0.243	0.224	0.262	0.263	0.246	0.280
*	-	-	-	-	-	-	-	Fracture of the sternum or ribs	1010	0.179	0.158	0.199	0.185	0.166	0.203
N21 Fracture of pelvis	41	0.207	0.136	0.279	0.207	0.143	0.271	Fracture of pelvis	906	0.194	0.172	0.216	0.205	0.185	0.225
N28 Moderate to severe TBI^	319	0.125ᵃ	0.109	0.141	0.138ᵃ	0.122	0.155	Severe traumatic brain injury^	715	0.184ᵃ	0.16	0.208	0.194ᵃ	0.172	0.217
N43 Internal haemorrhage in abdomen/pelvis	195	0.102ᵃ	0.079	0.124	0.103ᵃ	0.082	0.124	Abdominal or pelvic organ injury	668	0.161ᵃ	0.138	0.183	0.182ᵃ	0.162	0.203
N14 Other injuries of muscle & tendon/other dislocations	75	0.068	0.032	0.105	0.067	0.041	0.094	Muscle and tendon injuries	551	0.089	0.067	0.274	0.108	0.088	0.127
N37, N17, N18 Crush injury, fracture foot/hand bones	58	0.108	0.058	0.159	0.098ᵃ	0.06	0.136	Fracture of foot bones except ankle	477	0.168	0.143	0.193	0.179ᵃ	0.156	0.202
N40, N44 Contusion/open wound	68	0.071	0.038	0.104	0.081	0.049	0.113	Open wounds	258	0.110	0.075	0.146	0.133	0.100	0.165
N33, N34 Spinal cord lesion	38	0.244	0.169	0.32	0.281	0.201	0.361	Spinal cord lesion at neck level	238	0.316	0.265	0.366	0.333	0.287	0.379
N33, N34 Spinal cord lesion	38	0.244	0.169	0.32	0.281	0.201	0.361	Spinal cord lesion below neck level	179	0.356	0.300	0.411	0.373	0.322	0.424
N27 Minor TBI	314	0.101	0.087	0.114	0.104	0.091	0.116	Minor traumatic brain injury	170	0.068	0.029	0.106	0.100	0.062	0.138
N37, N17, N18 Crush injury, fracture foot/hand bones	58	0.108	0.058	0.159	0.098	0.060	0.136	Fracture of wrist and other distal part of hand	153	0.070	0.034	0.106	0.085	0.052	0.117
*	-	-	-	-	-	-	-	Fracture of skull	150	0.143	0.097	0.187	0.158	0.117	0.199
*	-	-	-	-	-	-	-	Fracture of face bone	135	0.140	0.087	0.194	0.150	0.104	0.196
*	-	-	-	-	-	-	-	Superficial injury	117	0.076	0.024	0.128	0.100	0.053	0.148
*	75	0.068	0.032	0.105	0.067	0.041	0.094	Dislocation of shoulder	109	0.110	0.059	0.16	0.136	0.087	0.184
*	75	0.068	0.032	0.105	0.067	0.041	0.094	Dislocation of hip	55	0.171	0.067	0.274	0.188	0.105	0.270
N8, N9, N10 Burns (including lower airways)	19	0.084	0.035	0.134	0.129	0.050	0.207	Burn covering ≥ 20% TBSA	55	0.156	0.077	0.234	0.176	0.100	0.251
N8, N9, N10 Burns (including lower airways)	19	0.084	0.035	0.134	0.129	0.050	0.207	Burn covering < 20% TBSA or unspecified	54	0.110	0.021	0.198	0.131	0.048	0.214
N8, N9, N10 Burns (including lower airways)	19	0.084	0.035	0.134	0.129	0.050	0.207	Lower airway burns	34	0.243	0.099	0.386	0.222	0.105	0.339
*	-	-	-	-	-	-	-	Nerve injury	31	0.191	0.078	0.305	0.215	0.140	0.326
N35, N36 Asphyxiation/Non-fatal submersion	6	0.277	0.120	0.433	0.249	0.086	0.411	*	-	-	-	-	-	-	-
Other	173	0.106	0.082	0.129	0.11	0.087	0.132	*	-	-	-	-	-	-	-

Adult weights taken from Gabbe BJ, Lyons RA, Simpson PM, Rivara FP, Ameratunga S, Polinder S, Derrett S, Harrison JE. Disability weights based on patient-reported data from a multinational injury cohort. Bulletin of the World Health Organization. 2016 Aug 31;94(11):806. *= Mapping not available as included in *Other* injury group in children and adolescents (refer Supplementary Table s2). ᵃ = Bonferroni adjusted *p* < 0.003 in Welch’s *t*-test. ^ Moderate to severe TBI combined in children and adolescents but separated in adults

**Fig. 2:**
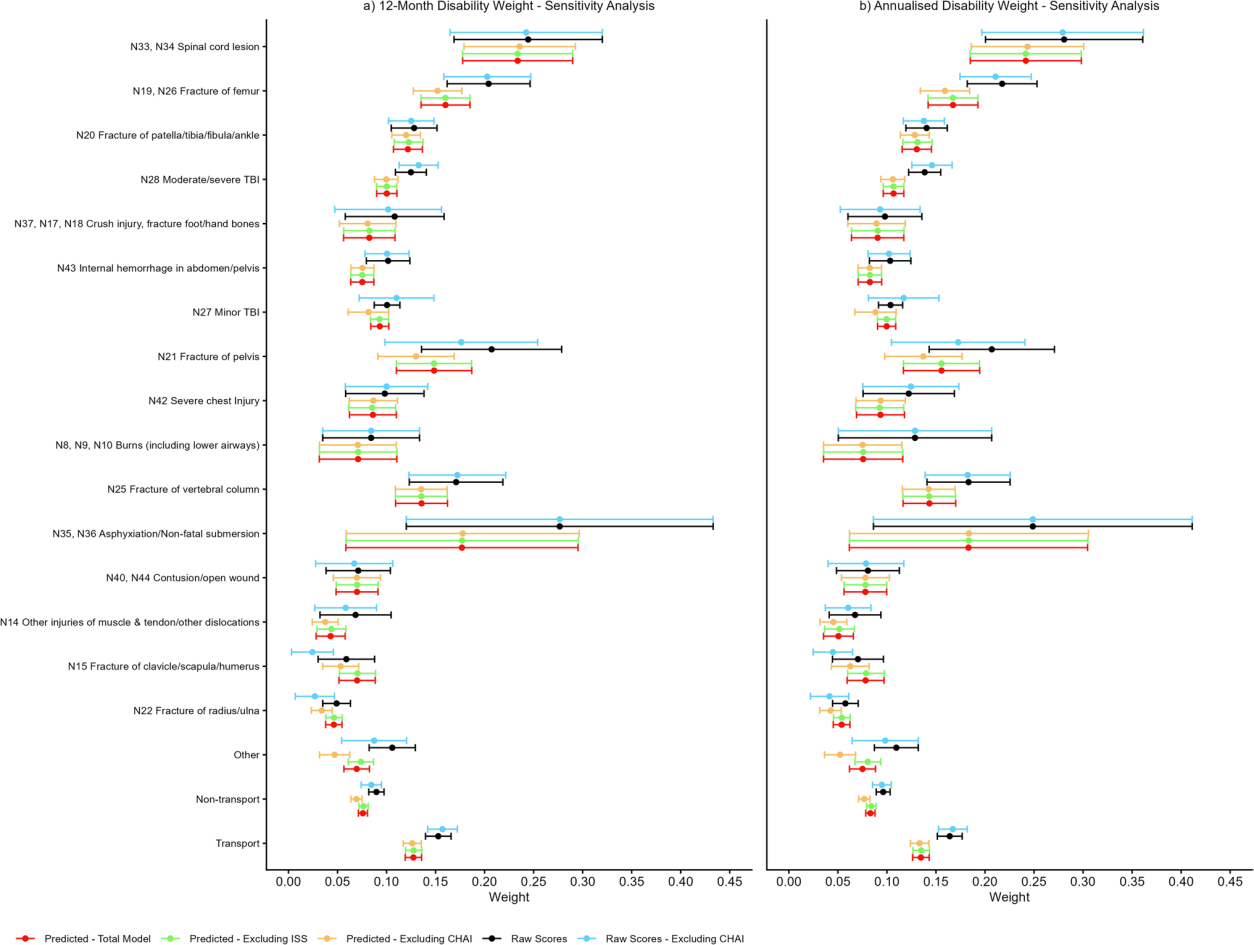
Sensitivity analysis for new disability weights for children and adolescents 5 to 17 years per GBD 2012 injury category

The sensitivity analysis revealed that using the predicted EQ-5D utility scores resulted in narrower 95% confidence intervals and excluding ISS in the modelled scores did not impact the results (Fig. 2). Most new disability injury weights were consistent across the five EQ-5D utility score types, but moderate to severe TBI and internal haemorrhage in abdomen/pelvis weights were lower using the predicted scores compared to the raw scores. Removal of the CHAI US cohort produced lower injury disability weights for fracture of pelvis; fracture of the clavicle/scapula/humerus; and fracture of radius/ulna. The degree of difference in the disability weights between moderate to severe TBI and minor TBI varied according to whether the raw or predicted utility scores were used in the calculation. The raw scores were higher for moderate to severe TBI, irrespective of if the CHAI cohort was included and the method for calculation of the utility scores.

The 12-month residual and annualised disability weights for children and adolescents for moderate to severe TBI, Fracture of clavicle/scapula/humerus and internal haemorrhage in abdomen/pelvis were lower than for adults (Table 3, Supplementary Fig. 3). The annualised disability weight for crush injury, fracture foot/hand bones in children and adolescents were also lower than for adults.

## Discussion

The establishment of disability weights pertaining to children and adolescents sustaining an injury is important but challenging in the context of the developing child. This study identified some variations in the calculations involving child and adolescent healthy norms from different countries. However, the patterns across estimated 12-month disability and annualised weights for injury were consistent, irrespective of the population norm used, supporting the use of a completely healthy norm as the base for the final weights.

Consistent with the adult disability weights, the 12-month weights are similar to the annualized weight for children and adolescents, making these weights potentially simpler to use. However, the annualized weight uses more of the information available, potentially more precisely represents the disability in the first 12 months after injury, rendering these weights potentially preferable.

This study found variation in the new disability weights for children and adolescents across injury, some of which differs to those used for adults [23]. The impact of applying adult disability weights to the calculation of YLD for children and adolescents has implications to the calculation of DALYs. Incorrectly applying higher adult disability weights to childhood YLD would artificially inflate the non-fatal YLD component of the DALY calculation (i.e. DALY = YLD + YLL (Years of Life Lost)). Conversely, incorrectly applying lower adult disability weights to childhood YLD would artificially deflate the non-fatal YLD component of the DALY calculation. The result of incorrect DALY calculations could potentially impact policy and resource allocations.

The lower 12-month residual and annualised disability in children compared to adults related to a fracture of clavicle/scapula/humerus and abdominal or pelvic organ injury may be due children’s bones and organs still developing and generally healing quicker. For example, fractures in children typically heal twice as fast and as they grow make for more easily remodelling of misaligned bones [36]. However, these differences between adults and children warrant further research. The higher new weights, both 12-month residual and annualised, for children and adolescents for spinal cord injury (SCI), femoral fracture, and pelvic fracture, were consistent with the disability weights for adults. These skeletal and spinal cord injuries reduce the physical functioning of children and adolescents which impacts on their independence in daily living, restricts their social interaction (e.g. via sporting activities) and school attendance and can increase the risk of mental health conditions such as anxiety or depression [37]. However, the 12-month residual disability attributed to pelvic fractures may also reflect the severity of associated injuries rather than the skeletal injury itself [38].

The final disability weights indicated higher disability weighting for children and adolescents who sustained a moderate to severe TBI than those with a mild TBI. This result was consistent with the adult disability weights, albeit a lower difference. However, the new disability weights for children who sustained a moderate to severe TBI and minor TBI varied according to if they were calculated from the raw utility scores or predicted scores, with differences between the two reduced for the weights calculated from the predicted scores. This has highlighted the challenges in capturing the important impacts on developmental trajectories and subsequent disability in children and adolescents related to TBI. The accurate assessment and identification of the severity of TBI in children and adolescents is complicated and challenging, potentially influenced by age with reliability lower in the young, with culture and health literacy found to affect care seeking and symptom reporting [39], health behaviours and use of preventive services [40]. This study does highlight that even mild TBI can have important impacts on developmental trajectories and subsequent disability. One explanation of the relatively high 12-month residual disability in children with mild TBI in our study is that children may miss out on being referred for services that children with moderate to severe TBI are offered [41, 42] and that these children and adolescents may have faced significant difficulties in accessing the essential medical, social, and financial services to aid their recovery in the first 12 months [43].

The high annualised than 12-month weights for children who sustained asphyxiation/non-fatal submersion potentially indicates a deterioration in health outcomes across 12 months for this cohort. Although the base in our study for this cohort was small (*n* = 6), the issues surrounding this injury warrants discussion. Children who sustain this type of injury experience poorer psychosocial health outcomes, with the potential that this type of injury may have consequences due to hypoxic brain injury. Poor outcomes have been associated with prolonged drowning (> 6 minutes) in children and post discharge morbidity can include issues of residual motor deficits, seizures [44], and neurocognitive outcomes in children following immersion include behavioural problems, poor communication, executive function and learning difficulties [45]. More research with a larger sample size is encouraged to validate these disability weights for this injury group in children and adolescents.

It was unsurprising that the new disability weights for the fracture of radius/ulna in children were consistent with adults. Although children’s bones and ligaments are still developing, with tendons impacting the hand/arm movement, the recovery is usually within six months for both children and adults.

The challenges in calculating disability weights in children and adolescents cannot be understated. The 12-month weights represent both residual disability at 12 months and the expected lifelong disability. However, variation within children and adolescent injury groups could potentially be hidden (e.g. heterogeneity, variability in treatments). As the 12-month annualised weights are time-adjusted to account for differences at each time point, they may not be an accurate representation of the burden of the injury (e.g. impacted by fluctuations at certain time points). The sparseness of certain injury groups in children and adolescents (i.e. asphyxiation/non-fatal submersion and burns (including lower airways)) challenges the precision of the disability estimates. The differences between raw and predicted weights, whilst marginal, were highest in the differences between the moderate/severe TBI and minor TBI disability weights. A sensitivity analysis was performed to see if a covariate in the original model impacted these differences. Whilst no such covariates were found, further research would be beneficial to reveal the nature of these differences. The lack of population norms for this cohort meant extensive sensitivity analysis, across a wide range of country population norms, was needed in this study. The lack of consistent healthy population norms for this cohort and lack of healthy norms spanning ages 5 to 17 (Supplementary Table s3) meant certain assumptions were required. Whilst this strengthened the final disability weights by confirming the patterns across injury and transport type, this warrants a call for further work in this domain. However, the sensitivity analysis performed in this study, across seven countries, showed consistent patterns across injury and transport type (Supplementary Figs. 1 to 2) and justified the decision to use perfect health (i.e. norm = 1) as the base population norm. The potential for linear predictive models, commonly used with EQ-5D prediction, has the potential to predict outside the EQ-5D boundary. Children and adolescents who are healthy prior to their injury may regain health states higher than the population norm, causing negative differences and forcing a decision to cap to zero. Major traumatic injuries that have long-lasting impacts, both physical and psychological, may mean that annualised disability weights are lower than 12-month disability weights due to the negative trajectory over this period. Further investigation is required into improving the calculation of disability weights in this cohort.

This study had several strengths. This study pooled a number of injury-specific and primary data of patient-centred outcomes at several of time points post injury, providing the ability to present disability weights covering the most common injury categories in the GBD 2013 and transport type in children and adolescents. The ability to directly compare the new children and adolescent disability weights to a number of adult disability weights provide new insights for researchers. The complexities of pooling data from multiple international data sets (e.g. mapping ICD-10; different collection of mechanism of injury; differing time points; mapping to the EQ-5D utility scores, etc.) meant that a small number of data points were excluded, but these were < 3% and considered acceptable [22, 46]. 

The disability weights for asphyxiation/non-fatal submersion and burns (including lower airways) were based on a small sample (i.e. *n* < 10 and *n* < 20 respectively). Consequently, the precision of the estimates (reflected by the wide 95% confidence intervals) should be interpreted with caution. This study calculated new disability weights following injury in high income countries, which may not align with low- and middle-income countries where the variation in health systems may impact the strength of these weights. However, the sensitivity analysis using seven different country norms may have alleviated this limitation. The accuracy of the coding of injury diagnoses cannot be guaranteed, and the collapsing of injuries into 17 GBD 2013 groups does lose granularity but allows the calculation for the weights. The shortcomings of the use of the ICD-10 for accurately describing childhood injuries may have influenced the accuracy of the injury diagnoses. For example, the use of ICD-10 has not been recommended as part of the new lexicon for re-characterising TBI in the United States National Institutes of Health work. Given the predominately adolescent age group in the cohort, it was possible that head injury with a normal CT scan and low GCS sustained in the context of intoxication has been erroneously coded as mod to severe TBI. The use of the ICD-10-CM “Unspecified” Codes for a head and brain injury in the Emergency Department (ED) settings has been highlighted by researchers [47], further questioning the accuracy of the ICD-10 for this injury. The EQ-5D and mapped PedsQL have several limitations when capturing developmental and psychosocial impacts in children and adolescents. The EQ-5D may have lacked developmental sensitivity for the children and adolescents in our pooled study. There is the possibility that the five measured dimensions (mobility, self-care, usual activities, pain/, anxiety/depression), and in some instances proxy responses to these questions, may have inadequately captured the nuanced developmental milestones and psychosocial functioning critical in our cohort. Finally, in children and adolescents, 12-month disability could depend on the nature of the injury, rendering it a limited proxy for lifelong disability. For example, fractures and intra-abdominal injuries in children heal relatively rapidly [36], whereas injuries such as TBI may be affected by the child’s normal developmental progress lasting beyond 12 months [48]. However, the 12-month residual and annualised weights generated in this study were chosen due to limited follow-up data available for this cohort and ability to compare with the adult weights.

## Conclusion

This study is the first to establish and reveal the importance of disability weights following injury specific to children and adolescents. The study highlights the complexities in calculating disability weights warrant further investigation into improved methods. As with adults, the findings of this study indicate that injuries in children and adolescents may impose long-term risks of disability and burden of disease estimates should reflect this.

## Supplementary Information

Below is the link to the electronic supplementary material.

ESM  Supplementary Material 1 (DOCX 315 KB)

## Data Availability

The data presented in this study are available on request from the relevant custodian. The data are not publicly available due to ethical and privacy issues.

## Abbreviations

BC: British Columbia
BCCH-LIO: British Columbia Children’s Hospital Longitudinal Injury Outcomes study
CHAI: Children’s Health After Injury study
CI: Confidence Interval
HRQoL: Health Related Quality of Life
ICD-9: International Classification of Diseases, 9th Revision
ICD-10: International Classification of Diseases, 10th Revision
OR: Odds Ratio
PedsQL: Pediatric Quality of Life Inventory
SD: Standard deviation
TBI: Traumatic Brain Injury
UK: United Kingdom
UKBOI: United Kingdom Burden of Injury study
VSTR: Victorian State Trauma Registry
