# Supplementary material for: Pediatric disability weights following injury based on patient-reported data from a multinational cohort
Source: Eur J Pediatr. 2026 Mar 21;185(4):197. doi: 10.1007/s00431-026-06845-2 (PMC13005863; doi:10.1007/s00431-026-06845-2)
Supplement: Supplementary file 1 — Supplementary Material 1 (DOCX 315 KB) [file 431_2026_6845_MOESM1_ESM.docx]

**SUPPLEMENTARY MATERIAL**

**Supplementary sTable 1**: Baseline Characteristics Children and Adolescents: Complete Versus Incomplete Follow-Up EQ-5D utility scores.

|  | **Incomplete Follow-up** | **Complete Follow-up** | **% Incomplete** | **Total** |
| --- | --- | --- | --- | --- |
|  | N=155 | N=1,817 | - | N=1,972 |
| **Age (Years) (Mean, SD)** | 13.9 (3.5) | 13.5 (3.5) | - | 13.6 (3.5) |
| **Age (Years) (Median, IQR)** | 15.0 (12.0-17.0) | 15.0 (11.0-16.0) | - | 15.0 (11.0-16.5) |
| **Age Group** |  |  |  |  |
| 5-9 years | 19 (12.3%) | 292 (16.1%) | 6.1% | 311 (15.8%) |
| 10-14 years | 41 (26.5%) | 569 (31.3%) | 6.7% | 610 (30.9%) |
| 15-17 years | 95 (61.3%) | 956 (52.6%) | 9.0% | 1,051 (53.3%) |
| **Sex** |  |  |  |  |
| Male | 115 (74.2%) | 1,331 (73.3%) | 8.0% | 1,446 (73.3%) |
| Female | 40 (25.8%) | 486 (26.7%) | 7.6% | 526 (26.7%) |
| **Cohort** |  |  |  |  |
| VSTR | 79 (51.0%) | 614 (33.8%) | 11.4% | 693 (35.1%) |
| VOTOR | 38 (24.5%) | 393 (21.6%) | 8.8% | 431 (21.9%) |
| CHAI | 9 ( 5.8%) | 570 (31.4%) | 1.6% | 579 (29.4%) |
| UKBOI | 5 ( 3.2%) | 116 ( 6.4%) | 4.1% | 121 ( 6.1%) |
| BCCH-LIO | 24 (15.5%) | 124 ( 6.8%) | 16.2% | 148 ( 7.5%) |
| **Transport Status** |  |  |  |  |
| Non-transport | 80 (51.6%) | 1,135 (62.5%) | 6.6% | 1,215 (61.6%) |
| Transport | 71 (45.8%) | 660 (36.3%) | 9.7% | 731 (37.1%) |
| Missing | * | 22 ( 1.2%) | * | 26 ( 1.3%) |
| **Injury Group** |  |  |  |  |
| N33, N34 Spinal cord lesion | * | 35 ( 1.9%) | * | 38 ( 1.9%) |
| N19, N26 Fracture of femur | 12 ( 7.7%) | 87 ( 4.8%) | 12.1% | 99 ( 5.0%) |
| N20 Fracture of patella, tibia, fibula, or ankle | 16 (10.3%) | 184 (10.1%) | 8.0% | 200 (10.1%) |
| N28 Moderate to severe traumatic brain injury | 28 (18.1%) | 291 (16.0%) | 8.8% | 319 (16.2%) |
| N37, N17, N18 Crush injury, fracture foot/hand bones | * | 54 ( 3.0%) | * | 58 ( 2.9%) |
| N43 Internal hemorrhage in abdomen or pelvis | 19 (12.3%) | 176 ( 9.7%) | 9.7% | 195 ( 9.9%) |
| N27 Minor TBI | 9 ( 5.8%) | 305 (16.8%) | 2.9% | 314 (15.9%) |
| N21 Fracture of pelvis | 8 ( 5.2%) | 33 ( 1.8%) | 19.5% | 41 ( 2.1%) |
| N42 Severe chest Injury | * | 37 ( 2.0%) | * | 41 ( 2.1%) |
| N8, N9, N10 Burns (including lower airways) | * | 15 ( 0.8%) | * | 19 ( 1.0%) |
| N25 Fracture of vertebral column | 6 ( 3.9%) | 66 ( 3.6%) | 8.3% | 72 ( 3.7%) |
| N35, N36 Asphyxiation, Non-fatal submersion | * | 6 ( 0.3%) | * | 6 ( 0.3%) |
| N40, N44 Contusion, open wound | 7 ( 4.5%) | 61 ( 3.4%) | 10.3% | 68 ( 3.4%) |
| N14 Other injuries of muscle & tendon and other dislocations | 6 ( 3.9%) | 69 ( 3.8%) | 8.0% | 75 ( 3.8%) |
| N15 Fracture of clavicle, scapula, or humerus | 6 ( 3.9%) | 69 ( 3.8%) | 8.0% | 75 ( 3.8%) |
| N22 Fracture of radius or ulna | 9 ( 5.8%) | 170 ( 9.4%) | 5.0% | 179 ( 9.1%) |
| Other | 14 ( 9.0%) | 159 ( 8.8%) | 8.1% | 173 ( 8.8%) |

Note: Completeness based on per cohort follow-up timing up to and including 12-months post injury.

**Supplementary sTable 2**: GBD2013 Injury Groups

| **GBD2013 Injury Group** |
| --- |
| N33, N34 spinal cord lesion |
| N19, N26 fracture of femur |
| N20 fracture of patella/tibia/fibula/ankle |
| N28 moderate to severe TBI |
| N37, N17, N18 crush injury, fracture foot/hand bones |
| N43 internal haemorrhage in abdomen/pelvis |
| N27 minor TBI |
| N21 fracture of pelvis |
| N42 severe chest injury |
| N8, N9, N10 burns (including lower airways) |
| N25 fracture of vertebral column |
| N35, N36 asphyxiation/non-fatal submersion |
| N40, N44 contusion/open wound |
| N14 other injuries of muscle & tendon/other dislocations |
| N15 fracture of clavicle/scapula/humerus |
| N22 fractur of radius/ulna |
| Other: this group included injuries such as amputation of one limb/toe, poisoning, injured nerves, environmental factors, dislocation of shoulder/hip/knee, fracture of ribs/sternum/skull/face bone, foreign body in ear/gastrointestinal or urogenital system, superficial injury, and injury to eyes. |

**Supplementary Table s3**: Published Population Pediatric Norms and Application to Current Study

| **Country & Authors** | **Setting** | **Published Norms** | **Used in Study** |
| --- | --- | --- | --- |
| Australia  Chen et al., 2015 | Online panel sample of Australian adolescents 11-17 years.  N = 2,020 | 11-14 years  15-17 years | 5-14 years  15-17 years |
| United Kingdom  McNamara et al., 2022 | 2017 &2018 waves of Health Survey for England (HSE).  N = 14,412 | By Male and Female:  16-17 years | By Male and Female:  5-17 years |
| Hungary  Inotal et al., 2024 | Hungarian central Statistical Office population representative survey on travelling habits of Hungarian people in 2022.  N = 11,910 | By Male and Female:  12-15 years  16-17 years | By Male and Female:  5-15 years  16-17 years |
| Indonesia  Fitriana et al., 2023 | Survey of children, recruited through the school, using stratified quota sample across 26 different schools located in different areas in Indonesia from July to December 2019.  N = 1,103 | 8-12 years  13-17 years | 5-12 years  13-17 years |
| Japan  Shiroiwa et al., 2021 | Random sample of children/adolescents 8-15 years and parents from 100 Japanese districts to complete door-to-door survey from January 2019 to March 2019.  N = 3,636 | By Male and Female:  8 years  9 years  10 years  11 years  12 years  13 years  14 years  15 years  16 years  17 years | By Male and Female:  5-8 years  9 years  10 years  11 years  12 years  13 years  14 years  15 years  16 years  17 years |
| China  Liang et al., 2024 | Multistage stratified random sample of children/ adolescents aged 9–17 from three cities of Jiangsu Province in China enrolled in the fourth grade of elementary school to the third grade of senior high school from September 2023 to November 2023.  N = 37,574 | By Male and Female:  9 years  10 years  11 years  12 years  13 years  14 years  15 years  16 years  17 years | By Male and Female:  5-9 years  10 years  11 years  12 years  13 years  14 years  15 years  16 years  17 years |
| Peru  Palacios-Cartagena et al., 2021 | Cell phone survey in school or extracurricular sports activities of children aged 12 to 18 years.  N = 1,229 | By Male and Female:  12 years  13 years  14 years  15 years  16 years  17 years | By Male and Female:  5-12 years  13 years  14 years  15 years  16 years  17 years |

**Supplementary Table s4:** New 12-Month and Annualised Disability Weights for Children and Adolescents (5-17 years) by Transport Status

|  |  | **12-Month Residual** |  |  | **Annualised** |  |  |
| --- | --- | --- | --- | --- | --- | --- | --- |
| **Transport Status** | **Base** | **Mean** | **95% Low** | **95% High** | **Mean** | **95% Low** | **95% High** |
| Non-transport | 1228 | 0.090 | 0.082 | 0.097 | 0.096 | 0.089 | 0.103 |
| Transport | 735 | 0.153 | 0.140 | 0.166 | 0.164 | 0.151 | 0.177 |

**
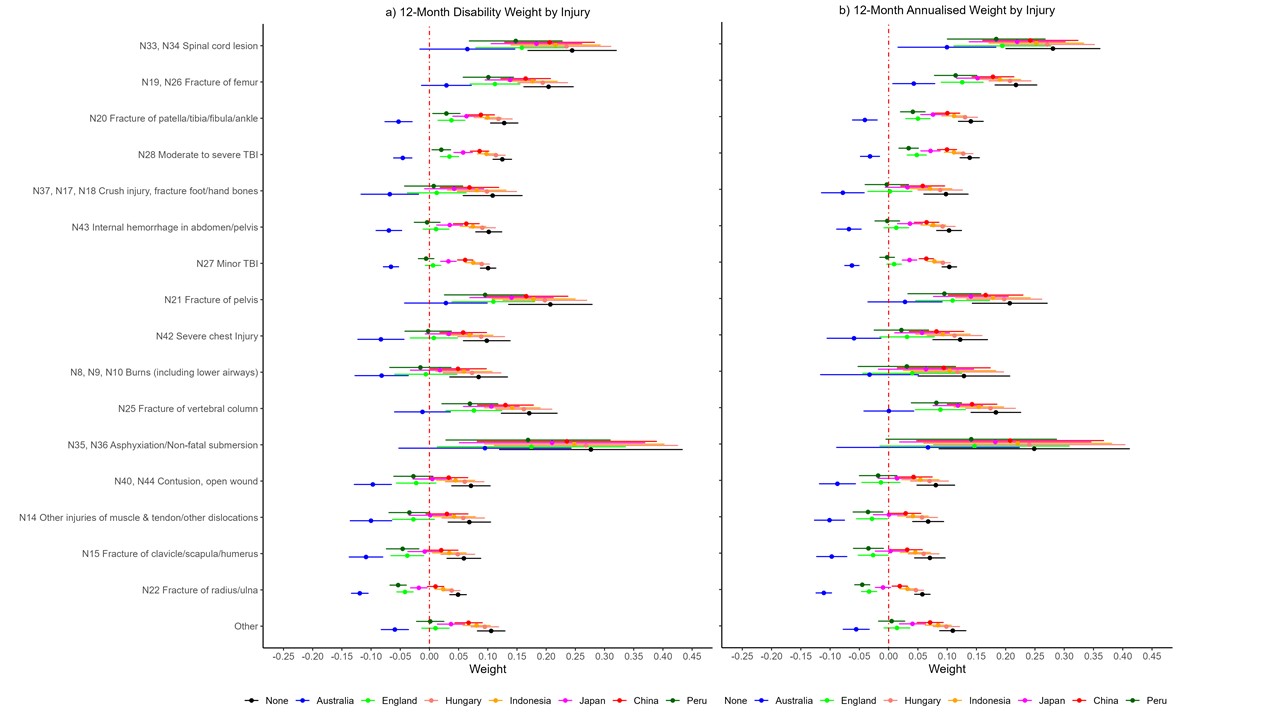
**

**Supplementary Figure 1:** New Disability Weights Per GBD 2013 Injury Category Per Country Norm

Note: None used 1-EQ5D.

**
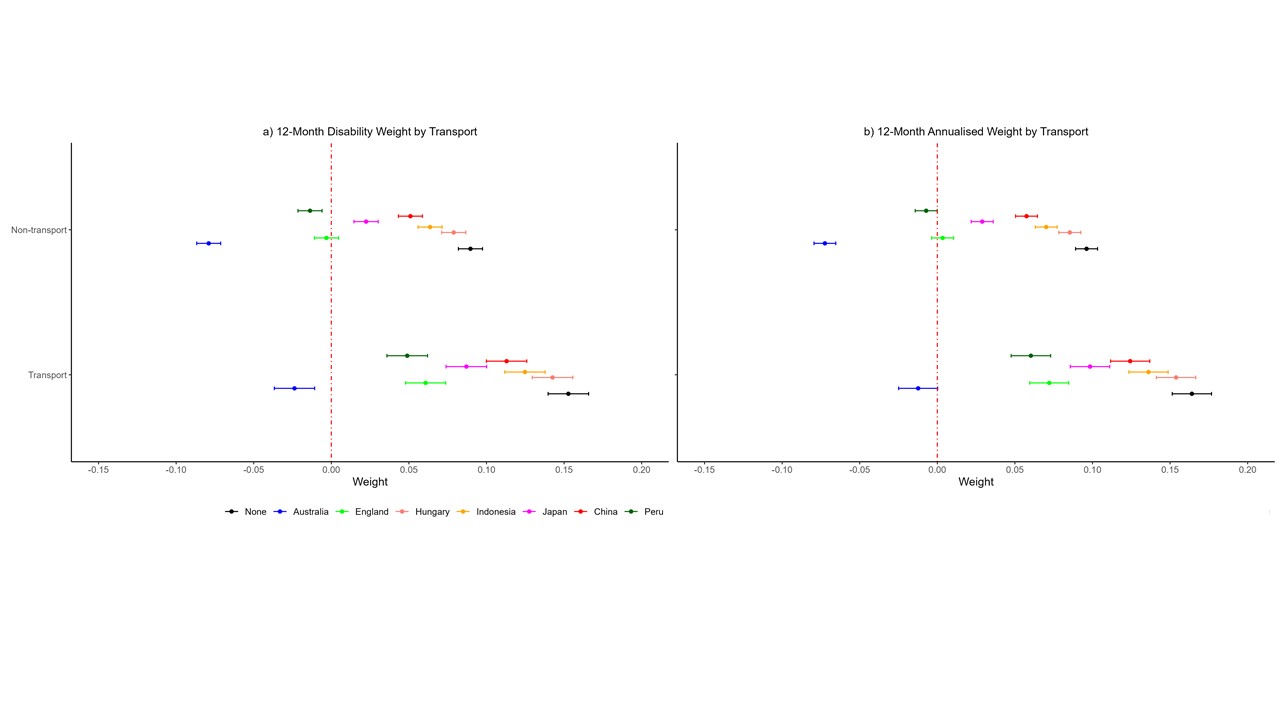
**

**Supplementary Figure 2:** New Disability Weights Per Transport/Non-transport Category Per Country Norm

Note: None used 1-EQ5D. Refer Supplementary Table s4 for new Disability weights per transport status.

**
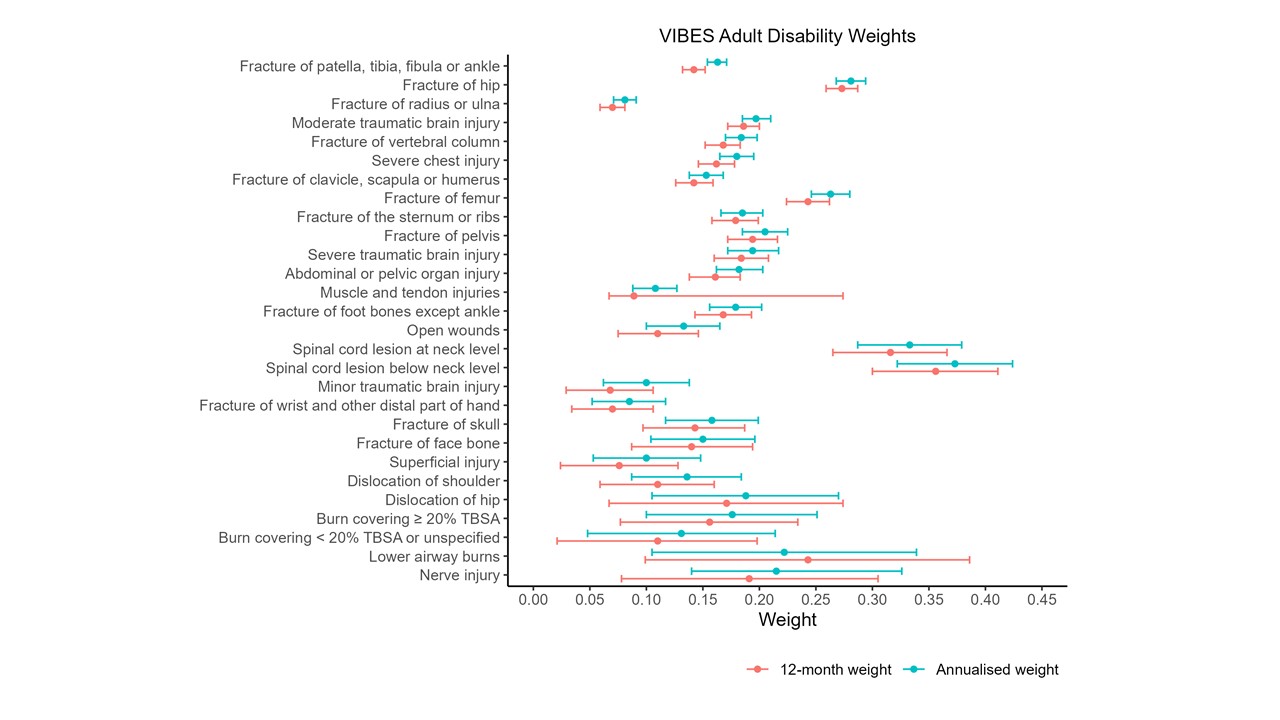
**

**Supplementary Figure 3:** VIBES Adult Disability Weights
